# Building implementation capacity in health care and welfare through team training—study protocol of a longitudinal mixed-methods evaluation of the building implementation capacity intervention

**DOI:** 10.1186/s43058-021-00233-7

**Published:** 2021-11-17

**Authors:** Hanna Augustsson, Veronica-Aurelia Costea, Leif Eriksson, Henna Hasson, Annika Bäck, Mårten Åhström, Anna Bergström

**Affiliations:** 1 Procome research group, Medical Management Centre, Department of Learning, Informatics, Management and Ethics, Karolinska Institutet, 171 77 Stockholm, SE Sweden; 2 Unit for implementation and evaluation, Center for Epidemiology and Community Medicine (CES), Region Stockholm, 171 29 Stockholm, SE Sweden

**Keywords:** Implementation science, Behavior change, COM-B, Implementation capacity, Knowledge translation, Process evaluation, Tailored implementation, Sustainability

## Abstract

**Background:**

To ensure the provision of high-quality safety and cost-effective health and welfare services, managers and professionals are required to introduce and ensure the routine use of clinical guidelines and other evidence-based interventions. Despite this, they often lack training and support in implementation. This project aims to investigate how a team training intervention, with the goal to build implementation capacity, influences participants’ implementation knowledge and skills, as well as how it influences implementation activities and implementation capacity within participating health and welfare organizations. Furthermore, the aim is to investigate how the organizations’ contexts influence the intervention outcomes.

**Methods:**

The building implementation capacity (BIC) intervention builds on the behavior change wheel, which considers implementation as a matter of behavior change. The intervention will be provided to teams of managers and professionals working in health and welfare organizations and seeking support to implement a guideline- or evidence-based intervention. The intervention consists of a series of interactive workshops that provides the participating teams with the knowledge and skills to apply a systematic implementation model. A longitudinal mixed-methods evaluation, including interviews, surveys, and document analysis, will be applied over 24 months. The normalization process theory measure will be used to assess how the intervention influences implementation activities in practice and implementation capacity in the teams and the wider organizations.

**Discussion:**

This project has an ambition to add to the knowledge concerning how to promote the uptake of research findings into health care by building implementation capacity through team training in implementation. The project’s uniqueness is that it is designed to move beyond individual-level outcomes and evaluate implementation activities and implementation capacity in participating organizations. Further, the intervention will be evaluated over 24 months to investigate long-term outcomes of implementation training.

**Supplementary Information:**

The online version contains supplementary material available at 10.1186/s43058-021-00233-7.

## Contribution to the literature

- Implementation of evidence-based interventions in health and welfare organizations require managers and professionals to change behavior. Despite this, training in implementation and the methods that best support behavior change in the workforce are scarce.
- This project will provide insights into how team training may support implementation activities in practice, as well as the building of implementation capacity.
- The study will measure processes and outcomes of the training concerning implementation of specific evidence-based interventions and implementation capacity.
- The study will evaluate implementation capacity 24 months after the training and, thereby, contribute to understanding of implementation training’s long-term outcomes.

## Background

To ensure high-quality, safety, and cost-effectiveness of services delivered by health and welfare organizations, managers and professionals are continuously required to implement changes (i.e., continuously achieving sustainable change in routine practice), such as introducing and ensuring the routine use of clinical guidelines and other evidence-based interventions (EBIs). Despite this, development of skills in implementation is usually not a part of managers’ and professionals’ professional education nor the trainings in which they participate as part of their continuous development [1, 2]. As a consequence, a need to build implementation capacity has been emphasized as an important part of increasing the uptake of research findings into health and welfare organizations [3]. Most implementation trainings have targeted researchers, doctoral- and master-level students [3–9], or individual professionals through university courses, webinars [5], or a combination of workshops and webinars [9]. However, there are still limited efforts to build evidence-based knowledge and skills in implementation among managers and professionals. Consequently, a need for capacity-building interventions aimed at professionals and managers has been identified [10, 11].

Leadership is considered a crucial factor in implementation [12–14], and managers have a key role in fostering a supportive implementation climate [15]. Line managers (i.e., the managers who work closest to the professionals responsible for providing direct services [16] are usually responsible for implementation in practice, as well as overseeing implementation [17]. However, implementation is a team effort [18, 19]. Thus, it has been suggested that training in implementation should be provided to teams of professionals alongside their managers, rather than targeting individuals [11].

The benefit of any training will ultimately depend on whether acquired knowledge, attitudes, and skills are transferred to job-related activities [20]. The literature on transfer of training has identified three large categories of factors that influence whether what is learned from training is transferred into behaviors. These include *trainee characteristics* (i.e., cognitive ability, self-efficacy, motivation, and perceived utility of training), *training design* (i.e., behavioral modelling, error management, and realistic training environments), and *work environment* (i.e., transfer climate, support, opportunity to perform, and follow-up) [21]. These factors are important to consider when designing and delivering training interventions. However, the providers of training interventions usually have little influence on the work environment where the learned skills are supposed to be transferred. This is problematic because even training programs that are designed and delivered effectively will fail to produce positive outcomes if the work environment does not encourage the use of targeted behaviors [21]. Support from managers and peers has been identified as one of the most important work environment factors to promote transfer of training [21]. Thus, team-training interventions provide an opportunity not only to improve the skills of the participating individuals but also to impact the environment where the implementation takes place.

Evaluations of training initiatives have often focused on individual-level outcomes, such as satisfaction with the training and improvements in knowledge and skills [11]. Although a few exceptions exist [e.g., [22]], long-term evaluations are scarce, and there is a lack of studies that have evaluated the impact on organizational outcomes. There is a need for longitudinal evaluations of training initiatives to increase the understanding of the extent to which training in implementation leads to sustainable outcomes [11].

The building implementation capacity (BIC) intervention is a team training intervention with the goal of improving work teams’ implementation knowledge and skills [23]. The intervention builds upon the idea that implementation concerns behavior change and proposes that implementation is more likely to be successful if tailored to the specific context in which change should happen [24, 25], as opposed to having generic implementation plans.

Aware that implementation is a reoccurring challenge, the BIC intervention strives to develop the team’s capacity to tailor implementation. Specifically, the intervention sets out to develop a set of dynamic capabilities (i.e., capabilities that relate to the organizations ability to manage change), which enables an organization to integrate clinical guidelines and other EBIs continuously and systematically, with the purpose of improving services and patient outcomes [26, 27]. The consequence is that participating teams should have the ability to concretize what the implementation implies in terms of mapping what behaviors need to change among which individuals and to develop an implementation plan with fit-for-purpose implementation strategies that align with the targeted individuals’ needs. One key difference between the BIC intervention and many other implementation-training efforts is that the participating organizations seek support in implementing something (the implementation case) relevant to them, and the BIC intervention’s focus is to build capacity for implementation. Thus, participating teams work with different implementation cases rather than focusing on a common implementation case.

An evaluation of the intervention’s first version [23] found positive outcomes in terms of participants perceiving the intervention as useful and relevant, and it increased participants’ knowledge about implementation. The evaluation provided further insights on how parts of the training were transferred to implementation activities in practice, as well as an understanding of how the BIC intervention could be enhanced. This input has now been used to improve the BIC intervention. Although the previous evaluation showed some impact on individuals’ self-reported knowledge and skills in implementation, it is still unknown if the intervention can impact the organizational capacities, what type of additional support organizations requires to integrate the use of the implementation model in their organizations, and what extent the implementation model is used in future implementations.

### Aim and research questions

This project aims to investigate how the BIC intervention influences participants’ implementation knowledge and skills, as well as how it influences implementation activities and implementation capacity within participating health and welfare organizations. Furthermore, the aim is to investigate how the organizations’ contexts influence the intervention outcomes.

The following research questions (RQ) will be examined.

1. To what extent does the BIC intervention increase participating teams’ implementation knowledge and skills?
2. What type of support is requested and provided to participating teams in addition to the BIC intervention?
3. How is the acquired knowledge from the BIC intervention transferred into implementation activities in practice?
4. How does the BIC intervention influence the organizational implementation capacity?
5. How do the participating organizations’ contexts affect their ability to achieve the intended implementation and apply the knowledge and skills gained through the BIC intervention in future implementations?

### Theoretical approach

The normalization process theory (NPT) will guide the evaluation of the BIC intervention. The NPT provides understanding of how practices become normalized and how work routines are created [28, 29]. In the current project, NPT will be used to understand the normalization of the clinical guidelines and EBIs on which the teams are working in their implementation cases, as well as the normalization of the BIC implementation model’s use as a tool to support future implementation. NPT is concerned with explaining what work people do, or need to do, with regard to implementing new practices, which is conceptualized in a set of four core constructs: coherence*,* which concerns the sense-making work that people do individually and collectively to operationalize new practices; cognitive participation, which mirrors the relational work that people do to build and sustain a community of practice; collective action, which is the operational work that people perform to enact a set of practices; and reflexive monitoring, which includes the appraisal work people conduct to assess and understand the ways that a new set of practices affects them and others. Due to its explanatory nature, NPT can be useful to assess the prerequisites for implementation, as well as to evaluate the progress of implementation. The application of NPT in this study will be operationalized using the normalization process theory measure (NoMAD) [30, 31].

## Methods

### Study design

The intervention evaluation will be conducted with a parallel convergent mixed-methods longitudinal study design [32] over 24 months. The evaluation’s focus will be on assessing changes in knowledge, skills, and normalization of the specific implementation on which the organizations are working, as well as normalization of the BIC implementation model use in future implementations (i.e., implementation capacity). A process evaluation will be conducted to investigate how contextual factors influence intervention outcomes. The evaluation will be divided into one basic and one profound part. The basic evaluation will include all participating teams, and the profound evaluation will include a subset of teams for a more in-depth investigation of how the training influences activities and implementation capacity in the organizations. The Template for Intervention Description and Replication (TIDieR) guideline [33] will be used to describe the intervention (Additional file 1), and the Consolidated Standards of Reporting Trials (CONSORT) extension for randomized pilot and feasibility trials [34] will be adapted according to existing recommendations [35] and used to report the intervention’s evaluation. The Consolidated Criteria for Reporting Qualitative Research (COREQ) checklist [36] will be used for reporting the qualitative data [36].

### Setting, participants, and recruitment

The project will be conducted in Region Stockholm and municipalities within Stockholm County. Region Stockholm is Sweden’s largest health care provider region, serving a population of more than two million. The region is responsible for the health care provided to its citizens, including primary care, acute hospital care, and psychiatric care. Stockholm County consists of 26 municipalities, which are responsible for social services. A regional research and development unit will provide the intervention.

The BIC intervention is offered to teams of professionals and managers. A team consists of a manager and two to five colleagues. Participating organizations are recruited through information and marketing of the intervention via different channels reaching organizations in Region Stockholm. Participation is voluntary, and teams choose to register for the intervention themselves. As the intervention is offered twice a year, participants are continuously included in the intervention. The study population will include organizations participating in the BIC intervention and providing informed consent to participate in the research project. We estimate that approximately 40 teams, with 120–200 participants in total, will take part in the intervention and provide data for RQs 1 and 2. A purposefully chosen subsample of these organizations will be included in a profound evaluation. The organizations will be chosen to achieve a maximum variation sample in terms of organization type and size, as well as a variation in implementation cases (i.e., the clinical guidelines or EBIs being implemented). In these organizations, staff not directly participating in the intervention will also be invited to participate in the study to assess implementation of the methods/guidelines on which the teams are working during the BIC intervention, as well as the implementation capacity in the organizations. We estimate that approximately 10 organizations will be included in the profound evaluation and contribute by responding to all stipulated RQs. As such, study participants include (1) participants attending the BIC intervention and (2) eligible staff at the participating organization (i.e., participants attending the BIC intervention together with their colleagues).

### The BIC intervention

#### Development

The intervention development started in 2013 and included a review of the literature on training initiatives in implementation science. This search provided information about implementation approaches with scientific support, including behavioral approaches (e.g., the behavior change wheel [BCW] [37]), strategies that were tailored to contextual barriers [38], and models for the implementation stages [39]. The literature was also searched for scientifically supported training designs. This search provided information that was used for developing the intervention’s pedagogical base, including the theory of experiential learning [40], the transfer of training research [21], and team learning [41, 42]. In a second step, interviews were conducted with local health-care stakeholders to investigate training needs and desired training outcomes, as well as contextual factors that influence opportunities to participate in training.

An intervention prototype was developed based on the information provided by the literature search and the interviews. This was discussed and revised in a workshop with national experts (i.e., researchers, consultants, and practitioners) in implementation, change management, and health and social care. A pilot test of the intervention in 24 teams provided information that was used to make improvements to the intervention design, mainly clarifications and simplification of the intervention content. The first version of the BIC intervention was evaluated in 2016–2017 [23]; and based on the results, further changes and refinements were made. These included clarifications of the content and materials used and the addition of practical training components to improve learning and to facilitate transfer of the acquired knowledge to implementation activities in practice.

#### Delivery

The revised BIC intervention consists of a series of four workshops (three hours each) over a period of approximately 3 months in which the participants will acquire evidence-based knowledge of implementation through short interactive lectures on implementation research, which are interspersed with the participants’ work with their own implementation case. The workshops will be delivered face-to-face in a large room where participants sit together with their own team (approximately 10 teams in total) to facilitate discussion and work with the implementation case. Between workshops, participants are expected to anchor the work they do within the BIC intervention at their workplace and collect comments on the work from colleagues. Participants will receive continuous feedback on their planning and implementation work from both workshop leaders and other participants during the workshops and between workshops. Approximately 3 months after the last workshop teams will be invited for an additional refill workshop where all steps in the implementation model are repeated. During this workshop, the teams will also have an opportunity to receive feedback and support on their current implementation phase and potential issues that have occurred. For an overview of the delivery and content in the workshops, see Additional file 2.

The intervention will be delivered by a group of professionals working in a research and development unit in Region Stockholm. The unit specializes in providing support concerning implementation and evaluation of EBIs to health and welfare organizations in the region. All workshop leaders are trained in implementation practice, as well as in the specific BIC-intervention model.

#### Content

The BIC intervention is based on an implementation model adapted from BCW [37] and determinants of practice [43]. The BCW approaches implementation as a matter of behavior change and provides a system for designing behavior change interventions [37]. When seen as a matter of behavior change, implementation normally indicates a new behavior should occur and, most often, an old behavior should cease. Therefore, the first step of the BCW is to understand the problem that the intervention aims to solve and, then, to choose and specify target behaviors of the intervention. Thereafter, a crucial part of the BCW is tailoring the intervention by analyzing what needs to change to enable the new behavior(s). At the core of the BCW lies the COM-B model, which is used to analyze what needs to change. The COM-B emphasizes that people need capability (C), opportunity (O), and motivation (M) to perform a behavior (B). In the BIC intervention, COM-B is complemented by Flottorp et al.’s [28] checklist for identifying determinants of practice. Based on an analysis of what needs to change, among individuals or in the environment, suitable implementation strategies (behavior change techniques) are finally identified, and their delivery planned [37, 44].

**Fig. 1:**
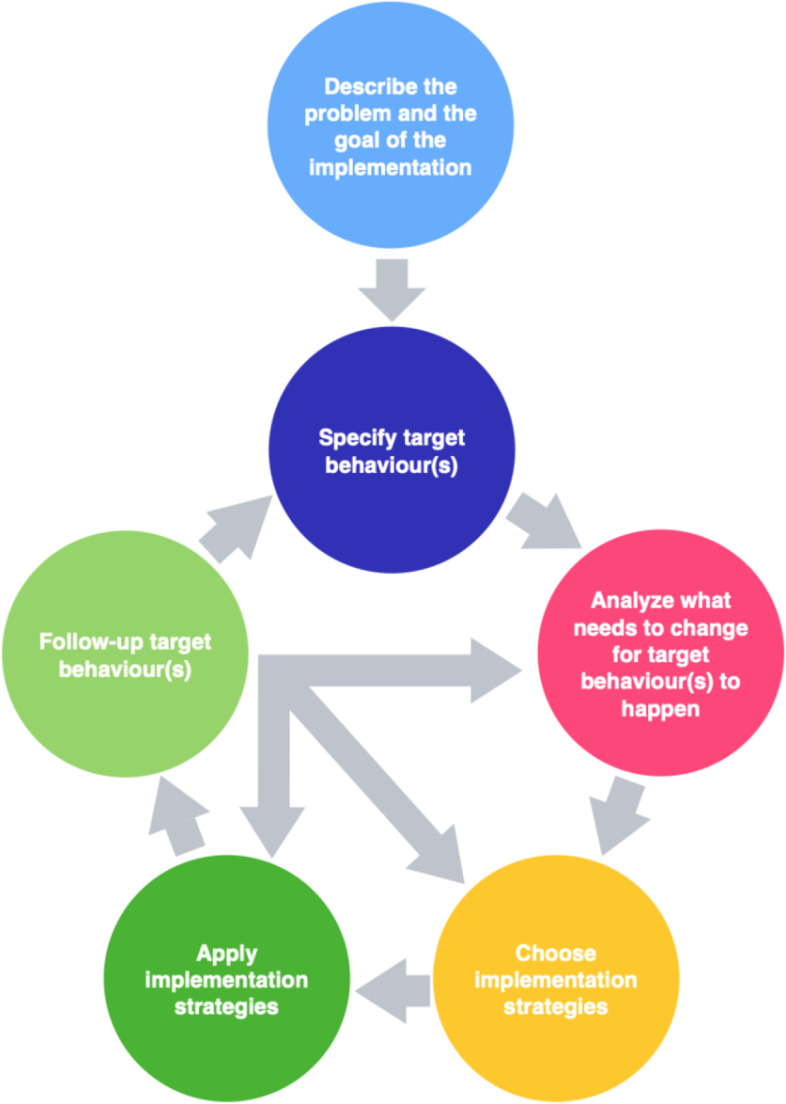
Graphic overview of the BIC-implementation model

The BIC implementation model (see Fig. 1 for a graphic overview of the implementation model) includes six steps in which participating teams should (1) describe what they wish to accomplish with the implementation; (2) identify and specify target behavior(s); (3) for each behavior, analyze what is needed for behavior change to occur (using the COM-B model and determinants of practice); (4) choose implementation strategies (e.g., education and reminders) based on the analysis in step 3; (5) apply implementation strategies; and (6) monitor occurrence of the target behavior (i.e., implementation fidelity). The arrows in the middle of the model highlight that monitoring target behavior will likely result in a need to go back in the process and refine the implementation plan. The implementation model’s steps and the activities to support the teams to learn these steps compose the core components of the intervention. Learning activities have been carefully designed to enable teams to achieve learning outcomes and, thus, also constitute core components of the BIC intervention. The use of implementation cases relevant to the teams and the continuous feedback that is provided by workshop leaders throughout the intervention allows tailoring of the intervention to teams’ specific needs.

All participants will receive a workbook in which the different steps are outlined. The workbook serves as a supporting document that didactically directs teams through the implementation model. The teams will also be provided with an implementation plan template that briefly outlines the steps in the implementation model. All material (in Swedish) is available upon request to the first author.

### Evaluation operationalization

Fidelity to the BIC intervention will be assessed by evaluating if the delivered intervention adheres to the content, frequency, duration, and coverage as described in the planned intervention [45]. The workshop leaders will keep notes about modifications of the planned intervention regarding changes in content, frequency, or duration of the training components as outlined in Additional file 2. Coverage will be assessed by taking notes on attendance for all participants in each workshop.

To examine the development of sustainable implementation capacity, the participating organizations will be followed for 2 years (20 teams) and 1 year (20 teams), respectively, because teams will be included in the intervention at different times starting from 2022 to 2023. As there are many teams participating in the BIC intervention, we will undertake two levels of data collection.

- *Basic evaluation* will cover all organizations participating in the BIC intervention. The purpose of the basic evaluation is to evaluate the extent to which participants increase their implementation knowledge and skills and understand their needs for further support in implementation (i.e., RQ 1 and 2).
- *Profound evaluation* will include 10 purposively selected organizations. These selected organizations will serve as case studies and provide a better understanding of how the BIC intervention works in different organizations, for example, in health care versus social service, in large versus small organizations, and at organizations working on a more strategic level versus those working more practice-oriented. This profound evaluation will include, in addition to the data collection occurring within the basic evaluation*,* a more in-depth evaluation of the extent to which learnings from the intervention are transferred to behaviors in practice, the organizations’ implementation capacities after attending the intervention, and the ways in which the organizational context influences these outcomes (i.e., RQ 3–5).

### Data collection tools and methods

The evaluation will be based on a combination of qualitative and quantitative methods, including surveys, individual interviews, focus group discussions, and document analyses. Below is a description of the different types of data that will be collected to answer each RQ. An overview of the data collection is shown in Table 1.

**Table 1:** Overview of the design of the evaluation including measures, respondents, and data collection

Outcome and process measures	Respondents	Data collection method	Time points	Level of evaluation
Implementation knowledge and skills	Intervention participants	Surveys measuring self-rated knowledge and skills	Baseline, post-intervention	Basic
Requested implementation support	Workshop leaders	Interviews and documents	3 and 6 months after the intervention	Basic
Use of acquired knowledge for implementation activities in practice	Intervention participants and their colleagues	S-NoMAD adapted to measure normalization of the implementation case Interviews and focus group discussions	Post intervention, 6 and 12 months after the intervention 6 and 18 months after the intervention	Profound
Implementation capacity	Intervention participants and their colleagues	S-NoMAD adapted to measure implementation capacity in terms of ability to apply the BIC implementation model Interviews and focus group discussions	6, 12, and 24 months after the intervention 6 and 18 months after the intervention	Profound
Understanding organizational context	Intervention participants and their colleagues	Individual interviews and focus group discussions	6 and 18 months after the intervention	Profound

*Implementation knowledge and skills (RQ1)* will be assessed at baseline and directly after the intervention using a survey [46] administered to all intervention participants (basic evaluation). In addition, knowledge, and skills will be evaluated at the team level to assess the extent to which participating teams are able to apply the BIC implementation model. The participating teams in the intervention are required to create plans for their implementation case and for fictive implementations. Their plans will provide information about how they will execute implementation of their implementation case. The fictitious cases will be used to assess participants’ knowledge in implementation and their ability to use the implementation model. This evaluation will provide an additional and more objective account of their learning, compared to the self-reported knowledge assessed through surveys.

#### Requested implementation support (RQ2)

Documentation outlining requested support outside the workshops will be collected through a structured logbook kept by the workshop leaders. These logbooks will provide information on extent and type of support requested by BIC intervention participants. To understand the need for additional support outside the BIC intervention workshops, individual interviews will also be conducted with workshop leaders.

#### Use of acquired knowledge for implementation activities in practice (RQ 3)

To assess how the acquired knowledge is transferred to implementation activities in practice, organization staff (i.e., intervention participants and their colleagues) included in the profound evaluation will be invited to respond to the Swedish version of the normalization process theory measure (S-NoMAD) [31]. S-NoMAD is designed for adaptation to the EBI being implemented. Thus, respondents will answer questions concerning the normalization of the specific implementation case that the teams work on during the BIC intervention. The measure will be conducted at three points: directly after the intervention and at 6- and 12-month follow-ups.

#### Implementation capacity (RQ4)

To assess how the BIC intervention influences organizational implementation capacity, organizational staff included in the profound evaluation will retake the S-NoMAD [31] now adapted so that the questions refer to the normalization of using the BIC implementation model (rather than the normalization of a specific implementation case). Measures will be conducted at 6, 12, and 24 months as follow-up.

To understand further how the implementation model taught in the BIC intervention has been used in the participating organizations—in relation to the implementation case that the teams have been working on during the intervention and other implementation efforts—individual interviews and focus group discussions will be conducted with intervention participants and eligible staff working at all participating organizations in the profound evaluation. The interviews will be conducted at two time points: 6 months and 18 months after completion.

#### Understanding organizational context (RQ5)

To explore how the participating organizations’ context affect their ability to achieve the intended implementation and apply the knowledge and skills gained through the BIC intervention in future implementations, individual interviews, and focus groups discussions will be conducted at 6 and 18 months follow-up. Informants will include participants and their colleagues from all the organizations included in the profound evaluation. The focus will be to understand under which circumstances the BIC intervention builds implementation capacity through an investigation of contextual factors.

### Data analysis

Qualitative data will be analyzed using qualitative content analysis [47] in the software NVivo. The quantitative analysis will include descriptive statistics, chi-square tests, and when appropriate, Fisher’s exact tests. Multilevel modeling will be used to assess changes over time because individuals are nested within teams. Data will be analyzed using R statistical software.

## Discussion

Although many empirical studies and theoretical frameworks highlight the importance of managers and staff for successful implementation, there is limited knowledge of how they can be supported and trained in conducting implementation practice. This project has the ambition to add to the knowledge concerning how to promote the uptake of research findings into health care by building implementation capacity through implementation training [3].

The project makes five main contributions to the research on training to build implementation capacity. First, manager and staff engagement and activities are crucial in implementation efforts. Therefore, the BIC intervention applies a team training approach. This is novel to implementation training, which has traditionally been delivered to individuals, for example, through university courses [3–9]. This project will provide insights into how team training may support implementation activities in practice, as well as the building of implementation capacity. Second, we aim to move beyond individual-level outcomes and evaluate how the BIC intervention influences implementation capacity and activities within the participating organizations. This will provide information on whether acquired knowledge and skills are transferred to implementation activities in practice.

Third, the project provides a scientific evaluation of a specific implementation case in the participating organizations. For this purpose, we will adopt a new, validated, and translated measurement: S-NoMAD [48]. The use of S-NoMAD will enable this evaluation, despite the participating teams having different implementation cases. Thus, the project will use an instrument for assessing impact of training initiatives across implementation cases and settings, which can provide useful information for future research and evaluations of training initiatives.

Fourth, evaluations of training interventions are often limited to short-term outcomes [11]. Consequently, the extent to which training interventions may lead to more sustainable outcomes, such as maintained implementation capacity, is not known. In addition, a 2-year follow-up focused on quantifying implementation capacity is, to our knowledge, uncommon. Therefore, we will use S-NoMAD to evaluate the sustained use of the implementation model, upon which the BIC intervention builds, to investigate the normalization of its use in the implementation of clinical guidelines and EBIs beyond the cases during the intervention. Measuring implementation capacity at different points in time will facilitate an understanding of the institutionalization of implementation capacity, here operationalized as the normalization of using the BIC implementation model. Last, one major problem in implementation is transferability between different contexts. The BIC intervention targets a wide range of different organizations, which, in combination with the investigation of influencing contextual factors, will contribute to understanding under which circumstances the BIC intervention can lead to implementation activities in practice and to increased organizational implementation capacity.

## Supplementary Information

**Additional file 1:.** The TIDieR (Template for Intervention Description and Replication) Checklist*: Information to include when describing an intervention and the location of the information

**Additional file 2:.** Content of each workshop in the BIC-intervention

## Data Availability

Transcripts from individual interviews and focus group discussions are not publicly available. However, data are available from the authors upon reasonable request, after ensuring that the integrity and confidentiality of respondents can be maintained. Quantitative data sets are available from the corresponding author on reasonable request.

## Abbreviations

BCW: Behavior change wheel
BIC: Building implementation capacity
COM-B: Capability, opportunity, motivation-behavior
EBI: Evidence-based intervention
NPT: Normalization process theory
S-NoMAD: Swedish version of the normalization process theory measure
