# Supplementary material for: Building implementation capacity in health care and welfare through team training—study protocol of a longitudinal mixed-methods evaluation of the building implementation capacity intervention
Source: Implement Sci Commun. 2021 Nov 17;2:129. doi: 10.1186/s43058-021-00233-7 (PMC8596934; doi:10.1186/s43058-021-00233-7)
Supplement: Supplementary file 2 — Additional file 2:. Content of each workshop in the BIC-intervention [file 43058_2021_233_MOESM2_ESM.docx]

## Additional file 2. Content of each workshop in the BIC-intervention

| **Workshop 1** | **Workshop 2** | **Workshop 3** | **Workshop 4** | **Refill Workshop** |
| --- | --- | --- | --- | --- |
| Introduction to implementation | Follow-up on home assignment | Follow-up on home assignment | Follow-up on home assignment | Step-by-step repetition of the implementation model |
| Introduction to the BIC implementation model | Repetition of the implementation model | Repetition of the implementation model | Introduction to sustained implementation and handling setbacks | Support and feedback from workshop leaders on current implementation challenges in the teams’ ongoing implementation cases |
| Introduction to the implementation model - step 1: Describe the problem and the goal of the implementation | Teamwork on own implementation case - step 2: prioritize behaviors | Introduction to tailored implementation activities | Cross unit work: apply the implementation model to a new fictive case with the purpose to repeat the model and train on applying it |  |
| Teamwork on own implementation case - step 1: Describe the problem and the goal of the implementation | Introduction of how to specify behaviors | Teamwork on own implementation case - step 4: Choose implementation strategies | Lecturer led exercise in full class: Application of the implementation model to a new fictive case |  |
| Exercise on defining goals | Teamwork on own implementation case - step 2: specify target behaviors | Introduction of how to evaluate implementation progress and model step 6: Follow-up target behavior | Practical exercise: lessons learned from the team training intervention |  |
| Introduction of behavioral perspective on implementation and implementation model step 2: Specify target behavior(s) | Exercise: barriers to implementation | Teamwork on own implementation case – step 6: Follow-up target behavior |  |  |
| Teamwork on own implementation case - step 2: List potential behavior(s) | Introduction to barriers to implementation | Team and individual exercise: present step 1-6 for a participant from another team |  |  |
| Team and individual exercise: present step 1 and step 2 for a participant from another team | Teamwork on own implementation case - step 3: Analyze what needs to change for target behavior(s) to happen | Preparing homework: getting feedback from colleagues on the preliminary implementation plan |  |  |
| Introduction of home assignment: inform colleagues about the gained knowledge | Team and individual exercise: present step 1-3 for a participant from another team. |  |  |  |
|  | Introduction of tailored implementation activities |  |  |  |
|  | Exercise on transfer of training |  |  |  |
|  | Preparing homework: getting feedback from colleagues on the preliminary implementation plan |  |  |  |
